# Editorial: The Uncanny Valley Hypothesis and beyond

**DOI:** 10.3389/fpsyg.2017.01738

**Published:** 2017-10-17

**Authors:** Marcus Cheetham

**Affiliations:** ^1^Department of Internal Medicine, University Hospital Zurich, Zurich, Switzerland; ^2^University Research Priority Program Dynamics of Healthy Aging, University of Zurich, Zurich, Switzerland; ^3^Department of Neuropsychology, Institute of Psychology, University of Zurich, Zurich, Switzerland

**Keywords:** uncanny valley hypothesis, robotics, computer animation, computer graphics, virtual reality, human likeness, anthropomorphic design

Progress toward realistic simulation of human appearance, behavior, and interaction in the fields of robotics and computer-graphics has been accompanied by interest in the *Uncanny Valley Hypothesis* (*UVH*) (Mori, 1970). The UVH posits that the use of anthropomorphic realism in the design of characters and objects (e.g., robots, prostheses) might have a counterproductive effect. Instead of enhancing subjective experience of the character or object, certain degrees of greater realism might unsettle the observer and induce a negative affective state. This state is marked by feelings of personal disquiet and a sense of strangeness (i.e., an uncanny effect). Mori did not develop the UVH further or subject his idea to empirical test. But concern as to the UVH' potential relevance for anthropomorphic design has given impetus to a new and evolving field of research (e.g., Hanson, 2006; MacDorman, 2006).

The UVH describes affective experience and the relationship between this and humanlike realism in simple terms. The simplicity serves well to express the general notion of a potential problem in anthropomorphic design. But the UVH is not a hypothesis in the scientific sense of an empirically testable statement. Early scientific enquiry into the UVH placed emphasis on developing experimentally more tangible renderings of the UVH. This can be seen in early exploratory testing, debate about the meaning and measurement of the UVH′s affective dimension, and theoretical considerations as to mechanisms potentially conducive to uncanny experience (e.g., Hanson, 2006; MacDorman, 2006; Bartneck et al., 2007; Seyama and Nagayama, 2007). In this research topic, the review, opinion, hypothesis and theory, and original research articles make reference to the early work.

Different lines of empirical enquiry into anthropomorphic realism and human behavior have built on the early work. These include the behavioral and physiological investigation of a range of perceptual, cognitive and affective mechanisms (e.g., Chaminade et al., 2007; Looser and Wheatley, 2010; Saygin et al., 2012). In this research topic, de Borst and de Gelder review behavioral and physiological evidence of differences and similarities in the response of subjects to the appearance and the affective and motor behavior of human-like compared with natural human stimuli. Understanding the pattern of responses to these perceptual categories is important. Much work has focussed on the affective experience of more or less realistic nonhuman stimuli (e.g., Tinwell and Grimshaw, 2009). However, the perception and experience of humanlike realism and related affect within the human category itself has received very little attention (Cheetham et al., 2011), even though this category forms the point of reference for the UVH and the proposed problem in anthropomorphic design.

Empirical support for the uncanny idea has been inconsistent. A number of specific reasons for this are addressed in different articles of this topic. Generally, some inconsistency will be inherent to the use of different conceptual, technical, and methodological approaches to define and operationalize humanlike realism and human experience and behavior. But there appears to be a growing convergence toward the observation, description, and explanation of the uncanny idea in terms of properties and mechanisms of perceptual and category information processing. This is reflected in the review article by Kätsyri et al. in this research topic. Kätsyri et al. consider inconsistent findings, review different conceptualisations of the uncanny effect, and report whether these conceptualisations find empirical support.

One particular line of enquiry, with a relatively brief history of investigation in the field of the UVH, has focussed on what might be generally referred to as the *categorization difficulty hypothesis*. This posits that subjective difficulty assigning a categorically ambiguous stimulus to the human or non-human category induces a negative affective state. Typically, this hypothesis has been examined using stimuli to represent the nonhuman and human categories of the UVH' dimension of human likeness (e.g., Burleigh et al., 2013). In their hypothesis and theory article, Ferrey et al. explore whether the experience of negative affect relates to cognitive mechanisms that subserve the processing of conflicting information from different perceptual categories irrespective of whether these categories contain perceptual features that specify human likeness. To date, the categorization difficulty hypothesis has been considered from a cognitive perspective in relation to various concepts, such as processing fluency (e.g., Yamada et al., 2013). In their opinion article, Schoenherr and Burleigh present a social-cultural perspective on the occurrence of negative affect in relation to low previous exposure to categorically ambiguous stimuli (i.e., an inverse mere-exposure effect).

Original research in the field of the UVH has made wide use of *ad-hoc* developed self-rating scales (e.g., Looser and Wheatley, 2010). With little exception (e.g., Ho and MacDorman, 2010), no psychometrically valid and reliable measures of affect have been applied in uncanny-related research. In original research of this topic, Cheetham et al. use well-established physiological and validated behavioral measures to examine affect in relation to the processing of nonhuman and human perceptual category information and of conflicting category information. To overcome the interpretational issues that have dogged the conceptualization of uncanny feelings in the UVH, affect is examined in terms of the primary orthogonal dimensions of affective experience (Russell, 1980).

While a typical human observer has everyday expertise in the extraction, processing, and interpretation of human perceptual and category information, the observer has comparably little or no such experience in the processing of newly designed humanlike exemplars and their human-specifying perceptual cues. This asymmetry between the nonhuman and human in perceptual and categorisation experience and knowledge and its influence on affective experience has received scant attention to date (Cheetham et al., 2011). In this topic, Cheetham et al. apply a perceptual discrimination paradigm to investigate this asymmetry in terms of differences within and between non-human and human perceptual categories in perceptual sensitivity to human-specifying information and examine the relationship between this and affect. Burleigh and Schoenherr use a category-learning paradigm to examine the modulatory influence on negative affect of acquired category structure (using perceptual categories based on non-human stimuli) and repeated stimulus exposure. Similarly, Złotowski et al. investigate the influence of repeated exposure to human-robot interaction on affective experience, in their case applying a live interaction paradigm. Generally, these studies focus on psychological factors that influence subjective experience of nonhuman and human stimuli. In contrast, Handžić and Reed focus on the systematic variation of multiple parameters of normal and abnormal patterns of gait and show how varying these parameters may be used to influence subjective experience. Finally, Destephe et al. study the impact of a range of factors, such as intensity of emotion expressed by whole-body robotic movement, in relation to different categories of subjective experience and affective determinants (e.g., attitudes).

The contributions to this topic provide a snapshot of current research in the field of the UVH. Set in the context of previous work, this snapshot suggests at least one emerging trend in research. Work to ascertain the general relevance of the uncanny idea for anthropomorphic design dominated the early years of uncanny research. A comparative interpretation of findings to date and inconsistencies between these suggests that an uncanny effect is not generalizable across different individuals, stimuli, situations, tasks, and time. As this topic indicates, research is shifting toward the development of a differentiated understanding of specifically when, under what conditions and why effects consistent with the uncanny idea occur. The development of this understanding requires research and active publication of a broad range of findings. These include robust findings that support the uncanny idea, indicate no effects, show effects contrary to the uncanny idea, and findings that do not replicate previously published findings. By broadening the perspective beyond the focus on negative affect, in the narrow sense of the UVH, this informative approach can contribute to the development of knowledge about anthropomorphic effects that are consistent or at variance with the uncanny idea, promote therefore a differentiated understanding of the relevance of the uncanny idea for anthropomorphic design, and help to accumulate applied knowledge about the effective use of variously realistic anthropomorphic features to induce, direct, maintain, and motivate subjective experience and behavior.

## Author contributions

The author confirms being the sole contributor of this work and approved it for publication.

### Conflict of interest statement

The author declares that the research was conducted in the absence of any commercial or financial relationships that could be construed as a potential conflict of interest.
